# Regulation of mammalian MOR-1 gene expression after chronic treatment with morphine

**DOI:** 10.3892/ijmm.2012.1132

**Published:** 2012-12

**Authors:** ROSE V. PRENUS, EBENS LUSCAR, ZHI-PING ZHU, RAMESH B. BADISA, CARL B. GOODMAN

**Affiliations:** College of Pharmacy and Pharmaceutical Sciences, Florida A&M University, Tallahassee, FL 32307, USA

**Keywords:** morphine, naloxone, μ-opioid receptor, real-time qRT-PCR

## Abstract

Morphine is an effective analgesic that acts by binding to the μ-opioid receptor (MOR) coded in the human by the OPRM1 gene. In the present study, we investigated the regulation of μ-opioid receptor (MOR-1) mRNA levels in all-trans-retinoic acid-differentiated SH-SY5Y human neuroblastoma cells under *in vitro* conditions with 10 μM morphine treatment for 24 h. In addition, we measured the MOR-1 levels in recombinant Chinese hamster ovary (CHO) cells, transfected with human μ-opioid receptor gene (hMOR) with 10 μM morphine treatment for 24 h. The isolated mRNA from these cells was subjected to real-time quantitative RT-PCR analysis to determine the regulation of μ-opioid receptor gene expression. It was observed that morphine treatment did not alter MOR-1 levels in undifferentiated SH-SY5Y cells compared to undifferentiated control cells. However, the MOR-1 levels in all-trans-retinoic acid-differentiated cells were significantly higher compared to the undifferentiated cells. Morphine treatment in differentiated SH-SY5Y cells caused significant downregulation of MOR-1 expression compared to the control cells. In the morphine-treated CHO cells, the hMOR-1 mRNA levels remained the same as the untreated control. Finally, pretreatment of SH-SY5Y cells with 10 μM naloxone, the antagonist of μ-opioid receptor, for 1 h significantly blocked the downregulation of MOR-1 mRNA levels with morphine treatment. These findings suggest that regulation of MOR-1 gene expression is cell-type specific after chronic morphine treatment and provide some evidence in the understanding of morphine tolerance.

## Introduction

Opium, the substance derived from the poppy plant, *Papaver somniferum*, has been used in medicine as an analgesic agent of pain reliever in different parts of the world for over 6000 years. Morphine is one of the highly potent and abundant alkaloids present in the opium, and is responsible for analgesic property. As early as eighteenth century, morphine was used in surgical procedures and pain management. The World Health Organization (WHO) recommended its use for controlling pain in cancer patients (1). Since morphine also produces euphoric feeling, it has become one of the highly abused drugs in the world currently. Long-term or chronic use of morphine is shown to associate with drug tolerance (2). Drug tolerance not only limits the use of morphine in clinical application but also involves tragic circumstances in drug addicts. *In vivo* studies indicated that morphine alters gene transcription in the brain (3) and spinal cord after acute and chronic administration. Previous studies have demonstrated that morphine induces long-term changes in neurons (4).

It is widely believed that the behavioral changes in drug addicts could be due to the altered gene expression in central nervous system (CNS). Studies demonstrated that μ-opioid receptor (MOR-1) is the primary site of action for morphine and the other most commonly used opioids (5,6). The process of morphine tolerance is very complex (7), but from the clinical point of view, it is important to understand the mechanism of its tolerance, because it may lead to treatment and prevention of opiate addiction. The MOR-1 gene expression is regulated at the level of DNA transcription or post-transcription. Since the short-term morphine treatment does not downregulate the MOR-1 receptor (8), in the present work, we studied the long-term chronic morphine treatment for drug tolerance mechanism on the regulation of MOR-1 in SH-SY5Y cells and CHO cells at the post-transcriptional level. In addition, we also investigated the effect of morphine on the regulation of MOR-1 receptor mRNA levels in the presence of receptor antagonist naloxone.

## Materials and methods

### Materials

Morphine sulfate, naloxone hydrochloride and all-trans-retinoic acid were obtained from Sigma-Aldrich^®^ (St. Louis, MO, USA). All other routine chemicals and reagents used were of analytical grade.

### Cell cultures

The human neuroblastoma cells (SH-SY5Y) were purchased from the American Type Culture Collection (Manassas, VA, USA). The recombinant Chinese hamster ovary (CHO) cells, transfected with human μ-opioid receptor gene (hMOR), were a kind gift from Dr Richard Rothman, NIDA-NIH Addiction Research Center (Baltimore, MD, USA). Both cell-types were maintained separately as adherent monolayer cultures. The SH-SY5Y cells were grown in the media without phenol-red, in a ratio of 1:1 mixture of Dulbecco’s modified Eagle’s medium (DMEM) and Ham’s F12 medium (Invitrogen, Molecular Probes, Eugene, OR, USA), with 2.5 mM L-glutamine, 0.5 mM sodium pyruvate, and 1200 mg/l sodium bicarbonate, supplemented with 10% FBS, penicillin (100 μg/ml) and streptomycin (100 U/ml). The recombinant CHO cells, transfected with hMOR-1 gene, were grown in the same media in a ratio of 1:1 as described above, containing phenol-red. The medium was supplemented with 10% FBS, penicillin (100 μg/ml) and streptomycin (200–250 U/ml). During experimental studies with CHO cells, the phenol-red free medium was employed, supplemented with all components as mentioned above. The cultures were maintained in an atmosphere of humidified air with 5% CO_2_ at 37ºC in an incubator.

### Differentiation of SH-SY5Y cells

The neuroblastoma cells (5×10^5^) were seeded in culture dishes in complete medium (30 ml), and allowed to grow until the cells reached 70–80% confluence. All-trans-retinoic acid (RA) was dissolved in 95% ethanol as a stock of 10 mM. A known volume of RA stock was added to the cultures to attain a final concentration of 10 μM (9). Control cells received an equal volume of the vehicle (0.1%). All culture dishes were incubated for 72 h continuously without further renewal of growth medium in the incubator.

### Treatments with morphine in SH-SY5Y cells

Morphine sulfate was dissolved in deionized water as a 10 mM stock and added to the cultures to achieve a final concentration of 10 μM (10). Control cells received an equal volume of the vehicle. In some of the experiments, the cells were pre-treated with 10 μM naloxone hydrochloride (MOR-antagonist) for 1 h, followed by treatment with 10 μM morphine sulfate for 24 h.

### Treatments with morphine in recombinant CHO cells

The CHO cells were seeded in culture dishes in complete medium devoid of phenol-red. To the cells, a known volume of morphine stock was added to the cultures to attain a final concentration of 10 μM (10). Control cells received an equal volume of the vehicle. All culture dishes were incubated for 24 h continuously without further renewal of growth medium in the incubator.

### RNA isolation

At the end of 24 h treatment, cells were washed three times with PBS to remove the drug compounds and the serum proteins. Then the cells were harvested using cell scrapers, and centrifuged at 1500 rpm for 3 min. The cell-pellets were re-suspended in 1 ml PBS, and transferred into eppendorf tubes, and subjected to centrifugation at 1000 rpm for 3 min. Finally, the pellets were homogenized in 1 ml TRIzol reagent with VirTishear polytron homogenizer (Virtis Company, Inc., Cardiner, NY, USA). Total RNA was extracted with chloroform and isopropanol according to the manufacturer’s instructions (Invitrogen, Carlsbad, CA, USA). Following ethanol precipitation, the vacuum-dried RNA was dissolved in 100 μl of DEPC-water. The quantity of total RNA was measured by the Nanodrop ND-1000 spectrophotometer (NanoDrop Technologies, Wilmington, DE, USA). RNA was subjected to DNAase treatment for 30 min at 37ºC using DNase Treatment and Removal Reagent (Ambion, Austin, TX, USA). The purified RNA with A260/A280 ratio of ≥1.8 was subsequently used for cDNA synthesis.

### cDNA synthesis

The cDNA synthesis was performed with an iScript cDNA synthesis kit (Bio-Rad, Hercules, CA, USA) using 10 μg of total RNA according to the manufacturer’s instructions.

### Data analyses

Data were presented as mean ± standard error of the mean (SEM). Differences between the means were compared by Student’s t-test. Statistical significance is ascribed for P<0.05. Curve fitting was conducted using GraphPad Prism 3.02 (GraphPad Software Inc., San Diego, CA).

## Results

### Morphological differentiation of SH-SY5Y cells

**Figure 1:**
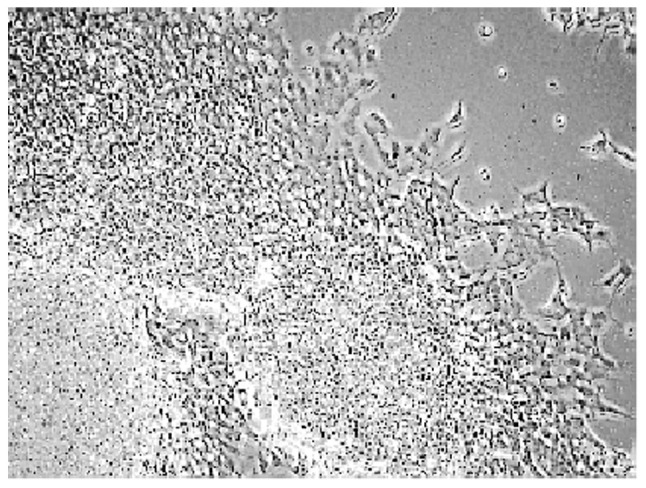

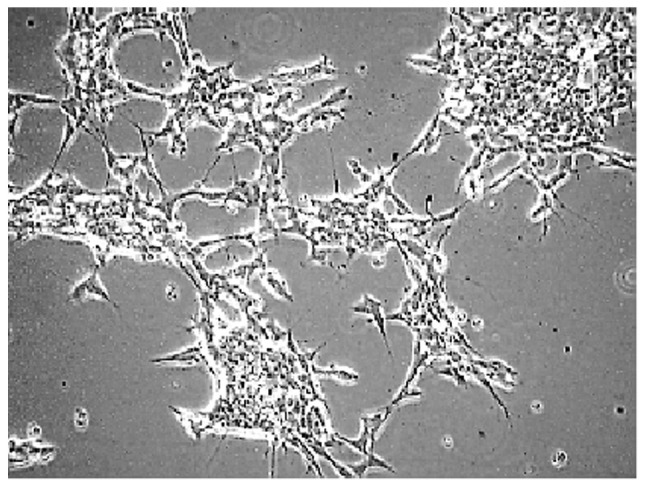
Morphology of undifferentiated and differentiated human SH-SY5Y cells. Cells were grown in culture dishes in the complete medium without RA (A) or with 10 μM RA (B). Unstained cells were photographed after 72 h of RA treatment using an inverted phase contrast IX-70 Olympus microscope. Neurite extensions in RA-induced differentiation (B) were seen clearly.

It was observed that the undifferentiated neuroblastoma cells grew mostly together in the form of clumps (Fig. 1A). On the other hand, cells treated with all-trans-retinoic acid (RA) for 72 h resulted in a significant cellular differentiation, characterized with elongated neurites (Fig. 1B). The concentration of RA was based on previous studies (10). Our results clearly demonstrate that RA promotes SH-SY5Y cell differentiation.

### Regulation of MOR-1 gene expression in SH-SY5Y cells

**Figure 2:**
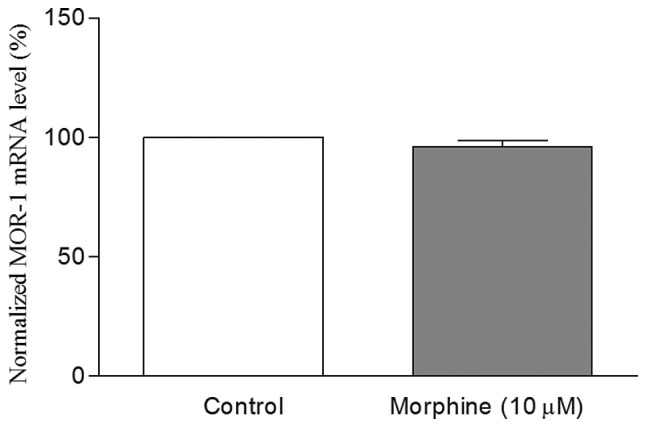
Effect of morphine on MOR-1 mRNA levels in undifferentiated SH-SY5Y cells. Cells were treated with 10 μM morphine for 24 h in the complete medium. MOR-1 relative mRNA levels were quantified by real-time PCR using β-actin as a reference gene. Data are presented as the mean ±SEM (n=3, ^*^P>0.05, insignificant in comparison to control, one-way ANOVA, Student’s t-test.

**Figure 3:**
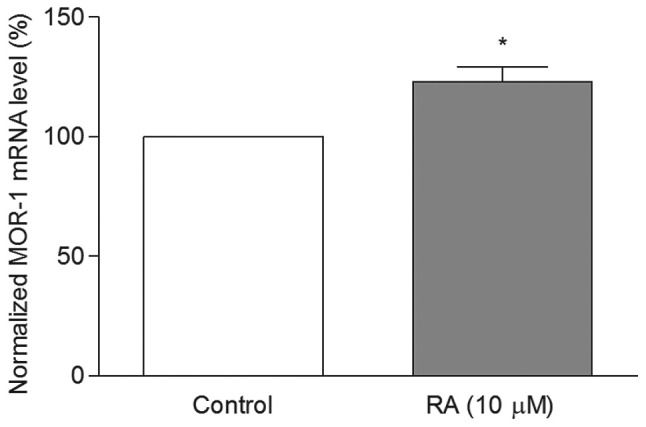
MOR-1 mRNA levels in undifferentiated (control) and differentiated SH-SY5Y cells. Cells were differentiated with 10 μM RA for 72 h. MOR-1 relative mRNA levels were quantified by real-time PCR using β-actin as a reference gene. Data are presented as the mean ± SEM (n=3, ^*^P<0.05, significant in comparison to control, one-way ANOVA, Student’s t-test.

**Figure 4:**
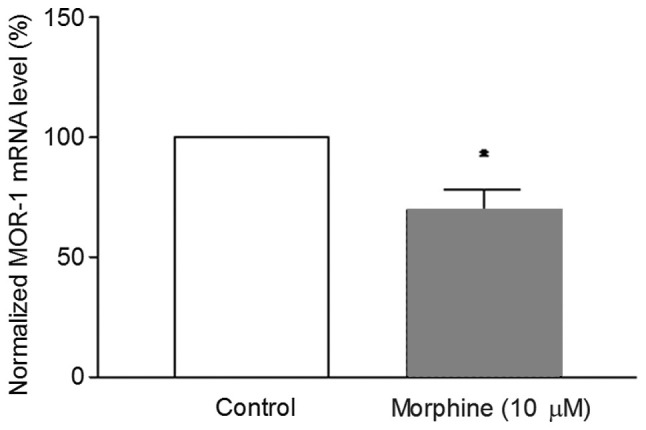
Effect of morphine on MOR-1 mRNA levels in differentiated SH-SY5Y cells. Cells were differentiated with 10 μM RA for 72 h, followed by treatment with 10 μM morphine for 24 h in the complete medium. MOR-1 relative mRNA levels were quantified by real-time PCR using β-actin as a reference gene. Data are presented as the mean ± SEM (n=3, ^*^P<0.05, significant in comparison to control, one-way ANOVA, Student’s t-test.

The mRNA levels of MOR-1 were quantitated by real-time RT-PCR using MOR-1 specific primers listed in Table I. β-actin, a housekeeping gene, was used for the normalization of gene expression. It was observed that the MOR-1 mRNA levels in undifferentiated cells with 10 μM morphine treatment for 24 h remained the same as those of the undifferentiated control cells (Fig. 2). However, in the RA differentiated cells, these levels were significantly increased in comparison to the undifferentiated control cells (Fig. 3). These results clearly show that the MOR-1 mRNA levels depend on the cellular differentiation. When the differentiated cells were treated with 10 μM morphine for 24 h, the MOR-1 mRNA levels were significantly reduced compared to differentiated control cells (Fig. 4). The results indicate that the MOR-1 gene regulation with morphine treatment depends on the cellular differentiation.

### Regulation of MOR-1 gene expression in hMOR-CHO cloned cells

**Figure 5:**
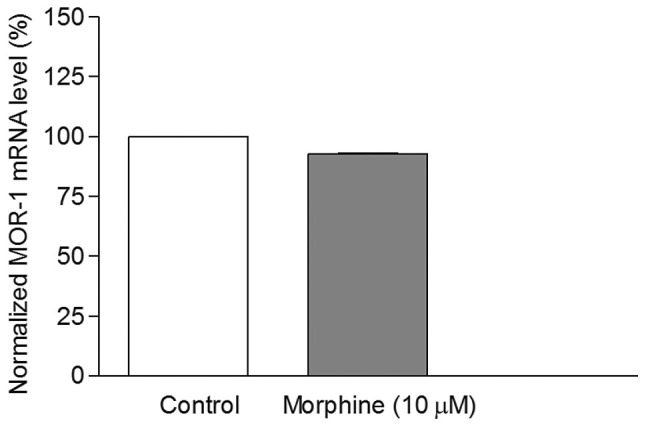
Regulation of MOR-1 mRNA levels in hMOR-CHO cells. Cells were treated with 10 μM of morphine for 24 h. MOR-1 relative mRNA levels were quantified by real-time PCR using β-actin as a reference gene. Data are presented as the mean ± SEM (n=3, ^*^P>0.05, insignificant in comparison to control, one-way ANOVA, Student’s t-test.

In an effort to understand the extent of MOR-1 gene regulation in a different cell system, we used Chinese hamster ovary (CHO) cells that were transfected with hMOR which stably express the MOR protein. The cells were treated with 10 μM morphine for 24 h, and the mRNA levels were measured by real-time RT-PCR. It was found that morphine treatment caused decrease in the mRNA levels (Fig. 5). However, this decrease was not significant (P>0.05). Therefore, the CHO cells were not utilized in our further studies.

### Reversal of MOR-1 mRNA downregulation by naloxone

**Figure 6:**
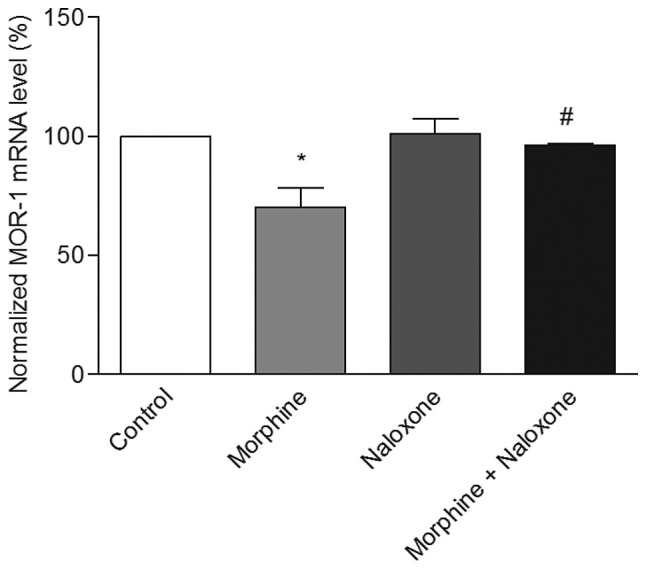
Reversal of MOR-1 mRNA downregulation by naloxone. SH-SY5Y cells were differentiated with 10 μM RA for 72 h in the complete medium, followed by pre-treatment with 10 μM naloxone for 1 h. Thereafter, these cells were treated with 10 μM morphine for 24 h. MOR-1 relative mRNA levels were quantified by real-time PCR using β-actin as a reference gene. Data are presented as the mean ± SEM (n=3, P<0.05: ^*^Significant in comparison to untreated control; ^#^Significant in comparison morphine treated cells, one-way ANOVA, Student’s t-test.

The effect of naloxone, which is an antagonist for MOR, was studied on the regulation of MOR-1 gene expression in morphine-treated cells. For this purpose, the differentiated human SH-SY5Y cells were pretreated with 10 μM naloxone for 1 h, followed by co-treatment with 10 μM morphine for 24 h. It was observed that morphine treatment caused a significant decrease in the MOR-1 mRNA levels, while naloxone alone did not alter these levels. However, in naloxone pretreated cells, morphine treatment did not decrease the MOR-1 mRNA levels (Fig. 6), and remained almost the same levels as those of the control. These results clearly show that naloxone reverses the morphine-induced downregulation of MOR-1 gene expression.

## Discussion

In the present study, the SH-SY5Y neuroblastoma cells were used as an *in vitro* model to investigate the effect of morphine on MOR-1 gene expression. This cell line was originally derived from neuroblastoma SK-N-SH clone, and expresses μ and δ receptors (11). Usually, the SH-SY5Y cells are differentiated by all-trans-retinoic acid (RA) to achieve neurite-outgrowth and morphological features (12). Both differentiated and undifferentiated SH-SY5Y cells were used as model cultures in neuroscience research (13–16). This posed a selection problem between differentiated and undifferentiated SH-SY5Y cells for our studies.

In order to find a suitable answer for this question, we first compared the MOR-1 gene expression levels in undifferentiated SH-SY5Y cells with morphine treatment. It was observed that morphine treatment did not alter the MOR-1 mRNA levels in these cells compared to undifferentiated control cells (Fig. 2). However, the levels were significantly upregulated in RA differentiated SH-SY5Y cells compared to the undifferentiated cells (Fig. 3). The results highlight that the process of differentiation appears to modulate the response to morphine treatment. These observations were consistent with previous report, where MOR-1 mRNA levels were shown to upregulate in RA differentiated SH-SY5Y cells (17). Since the MOR-1 levels were higher in the differentiated cells than the undifferentiated cells, we preferred to differentiate the cells with RA for further studies.

The morphological features of differentiated cells clearly showed that the cells have elongated neurite extensions (Fig. 1), which are in agreement with previous reports (18,19). We next studied the effect of morphine on MOR-1 mRNA levels in the differentiated cells. It was found that morphine downregulated the MOR-1 levels significantly (Fig. 4). The downregulation of MOR-1 with morphine treatment was also observed earlier in different cell lines (20–22).

We further studied the effect of morphine in recombinant CHO cells for MOR-1 mRNA levels. Morphine treatment did not alter the mRNA levels significantly in these cells (Fig. 5). The results clearly suggest that regulation of MOR-1 gene expression is cell-type specific. Earlier studies on recombinant CHO cells confirmed our results in terms of having no alteration in mu opoid receptor protein with morphine treatment (23).

Since morphine treatment caused downregulation of MOR-1 mRNA levels in our study, we investigated the compounds that act as antagonist to MOR-1 receptor to prevent the downregulation of MOR-1 gene. Naloxone, an opioid antagonist, was employed in our studies with 1 h pretreatment, prior to morphine treatment. It was observed that naloxone-pretreatment blocked the downregulation of MOR-1 gene expression significantly (Fig. 6). Similar observation was reported earlier, where naloxone was shown to block the downregulation of receptor protein with morphine treatment (10).

In conclusion, chronic morphine treatment caused the downregulation of MOR-1 gene expression in human differentiated SH-SY5Y cells, while naloxone reversed this process. The results clearly demonstrate that antagonists have a potential role in the treatment against morphine drug addiction.

## Figures and Tables

**Table I:** Sequence of the primers used in real-time PCR for human SH-SY5Y cells.

mRNA	Primers
MOR-1	F: 5′-ATGCCAGTGCTCATCATTAC-3′ R: 5′-GATCCTTCGAAGATTCCTGTCCT-3′
β-actin	F: 5′-GATGAGATTGGCATGGCTTT-3′ R: 5′-CACCTTCACCGTTCCAGTTT-3′

## Abbreviations

CNS: central nervous system
FBS: fetal bovine serum
MOR-1: μ-opioid receptor
PBS: phosphate-buffered saline
RA: all-trans-retinoic acid
WHO: World Health Organization
